# GIHP: Graph convolutional neural network based interpretable pan-specific HLA-peptide binding affinity prediction

**DOI:** 10.3389/fgene.2024.1405032

**Published:** 2024-07-10

**Authors:** Lingtao Su, Yan Yan, Bo Ma, Shiwei Zhao, Zhenyu Cui

**Affiliations:** ^1^ Shandong University of Science and Technology, Qingdao, China; ^2^ Shandong Guohe Industrial Technology Research Institute Co. Ltd., Jinan, China; ^3^ Qingdao UNIC Information Technology Co. Ltd., Qingdao, China

**Keywords:** HLA-peptide binding, model interpretation, GCNN, immunotherapy, affinity prediction

## Abstract

Accurately predicting the binding affinities between Human Leukocyte Antigen (HLA) molecules and peptides is a crucial step in understanding the adaptive immune response. This knowledge can have important implications for the development of effective vaccines and the design of targeted immunotherapies. Existing sequence-based methods are insufficient to capture the structure information. Besides, the current methods lack model interpretability, which hinder revealing the key binding amino acids between the two molecules. To address these limitations, we proposed an interpretable graph convolutional neural network (GCNN) based prediction method named GIHP. Considering the size differences between HLA and short peptides, GIHP represent HLA structure as amino acid-level graph while represent peptide SMILE string as atom-level graph. For interpretation, we design a novel visual explanation method, gradient weighted activation mapping (Grad-WAM), for identifying key binding residues. GIHP achieved better prediction accuracy than state-of-the-art methods across various datasets. According to current research findings, key HLA-peptide binding residues mutations directly impact immunotherapy efficacy. Therefore, we verified those highlighted key residues to see whether they can significantly distinguish immunotherapy patient groups. We have verified that the identified functional residues can successfully separate patient survival groups across breast, bladder, and pan-cancer datasets. Results demonstrate that GIHP improves the accuracy and interpretation capabilities of HLA-peptide prediction, and the findings of this study can be used to guide personalized cancer immunotherapy treatment. Codes and datasets are publicly accessible at: https://github.com/sdustSu/GIHP.

## 1 Introduction

HLA also known as MHC (major histocompatibility complex) molecules, are responsible for presenting peptides derived from intracellular or extracellular proteins to T cells. It is a crucial step in understanding and predicting immune responses, such as antigen presentation and T-cell activation (Kallingal et al., 2023). HLA molecules are classified into two major classes: class I and class II. Each class has different subtypes, and their binding abilities vary depending on the specific HLA subtype. For HLA class I, the open binding groove close to both ends restrict the size of the bounded peptides between 8–12 residues, whereas HLA class II incorporates peptides of length 13–25 residues (Wang and Claesson, 2014). As a results, existing methods can be classified into allele-specific and pan-specific methods. Allele-specific methods focus on predicting the binding affinity between a specific HLA allele. Pan-specific methods aim to predict HLA-peptide binding in a more general way, without the need for allele-specific training data. (Gizinski et al., 2024).

Allele-specific methods train separate models for each MHC allele and make predictions for individual alleles. NetMHC (Lundegaard et al., 2008) is a widely used allele-specific method, which utilize machine learning algorithm to learn the relationship between peptide sequences and their binding affinities to specific MHC alleles. NetMHC 4.0 (Andreatta and Nielsen, 2016) is also a sequence-based allele-specific method, which uses both BLOSUM62 and sparse encoding schemes to encode the peptide sequences into nine amino acid-binding cores. In comparison with the HLA (around 360aa in length), peptides length are much shorter, and such methods must take insertion methods to reconcile or extend the original sequence. In addition, deep learning-based methods have also been developed for MHC-peptide binding prediction. DeepMHCII (You et al., 2022), which utilizes deep convolutional neural networks (CNNs) to capture complex sequence patterns and interactions between peptide and MHC class II molecules. It takes the peptide and MHC protein sequences as input and uses multiple layers of convolutional filters to extract features from the sequences. These filters scan the input sequences at different lengths, capturing both local and global patterns. The extracted features are then fed into fully connected layers to make predictions of the binding affinity. MHCAttnNet (Venkatesh et al., 2020) utilizes a combination of bidirectional long short-term memory (Bi-LSTM) and attention mechanisms to capture important features and dependencies in MHC- peptide interactions. The Bi-LSTM processes the sequences in both forward and backward directions, capturing the dependencies and context in the data. The attention mechanism allows the model weight different parts of the input sequences based on their relative importance. This enables the model to focus on the most relevant regions of the peptide and MHC sequences during the prediction process. SMM-align (Nielsen et al., 2007) utilizes structural and sequence-based features to predict binding affinities for MHC class I alleles. It employs a PSSM alignment algorithm to align target peptide sequences with known binders and derive binding predictions. MHC-NP (Giguere et al., 2013) also incorporate structure with sequence-based features and employs a random forest regression model to make predictions. Allele-specific methods are particularly useful when the focus is on specific alleles of interest, allowing for more accurate predictions tailored to those specific alleles. However, developing and maintaining separate models for each allele requires a significant amount of experimental binding data and computational resources.

On the other hand, pan-specific methods have the advantage of predicting binding affinities not only for alleles present in the training data but also for new, unseen alleles. NetMHCpan and NetMHCIIpan (Reynisson et al., 2020) are widely used pan-specific methods. They take sequence feature as input, utilizes artificial neural networks (ANNs) to learn the relationship between peptide sequences and their binding affinities to MHCs. They consider various sequence-based features, including amino acid composition, physicochemical properties, and binding motifs. In comparison with these two methods, another pan-specific method MHCflurry (O'Donnell et al., 2018; O'Donnell et al., 2020) integrates additional information, such as peptide processing predictions and binding affinity measurements from mass spectrometry-based experiments, to enhance its predictions. Some sequence-based methods, such as BERTMHC (Cheng et al., 2021), leverage the power of the BERT language model to improve their performance. The BERT language model is pre-trained on a vast corpus of text data, which enables it to capture intricate patterns and dependencies within input sequences effectively. One of the advantages of using BERT for encoding peptide sequences is its ability to capture long-range dependencies and contextual information. This is particularly important in MHC binding prediction, where specific amino acid positions within a peptide can significantly affect the binding affinity. Because structure determines the function of proteins, therefore, some methods also incorporate structure information into their predictions. MixMHCpred-2.0.1 (Gfeller et al., 2018) employs a deep learning architecture capable of learning complex patterns and relationships between peptide sequences and MHC binding affinities. The model is trained on a diverse set of MHC alleles and covers a wide range of peptide lengths. This allows it to make accurate predictions for a broad range of MHC-peptide combinations. NetMHCpan-4.0 (Jurtz et al., 2017) utilizes a combination of structural and sequence-based features. It incorporates information from MHC-peptide complex structures and uses a machine learning approach to make pan-specific predictions. RPEMHC (Wang et al., 2024) is a deep learning approach that aims to improve the prediction of MHC-peptide binding affinity by utilizing a residue-residue pair encoding scheme. In RPEMHC, the peptide sequence and MHC binding groove are encoded as one-hot vectors, representing each amino acid residue and its position. AutoDock is a widely used molecular docking software that can be employed for MHC-peptide binding prediction. It uses a Lamarckian genetic algorithm to explore the conformational space and predict the binding modes and affinities of peptides within the MHC binding groove. By modelling the docking between the HLA protein and peptide ligands these methods have achieved accurate binding prediction performance. However, docking methods rely on sampling different conformations of the peptide and MHC molecule to find the best binding pose. However, the conformational space of peptides and MHC molecules can be vast, and exhaustively sampling all possible conformations is computationally infeasible.

In fact, no matter allele-specific or pan-specific methods, they all can be broadly categorized into two main categories: sequence-based and structure-based methods. Sequence-based methods utilize machine learning techniques to capture the sequence motifs and physicochemical properties important for HLA-peptide binding. These methods employ various algorithms, such as support vector machines (SVMs), random forests, or ANNs, to learn the relationships between peptide sequences and binding affinities from large datasets. Sequence-based methods have the advantage of being computationally efficient and applicable to a wide range of HLA alleles and peptides. Structure-based methods leverage the three-dimensional structures of HLA molecules and peptides to predict binding affinities. Molecular docking algorithms, such as AutoDock, are commonly used to explore the conformational space and calculate binding energies. These methods require knowledge of the 3D structures of the HLA molecule and peptide, limiting their applicability to cases where experimental structures are unavailable. Recent advancements in deep learning, such as CNNs and recurrent neural networks (RNNs), have shown promise in HLA-peptide binding affinity prediction. Deep learning-based methods can effectively capture complex sequence patterns and structural features, leading to improved prediction accuracy (Wang et al., 2023). These models often incorporate encoding schemes to represent peptide sequences or structural features and are trained on large datasets to learn the relationships between sequences and binding affinities. Despite notable progress, HLA-peptide binding affinity prediction still faces challenges and have some limitations. First, deep learning models are often considered as black boxes, meaning they lack interpretability. It can be challenging to understand the specific features or patterns that contribute to the model’s predictions. Interpretability is crucial in immunology research to gain insights into the molecular mechanisms underlying MHC-peptide interactions and to guide experimental studies; Second, existing methods often rely on sequence-based encoding schemes due to the limited availability of experimentally determined 3D structures for HLA-peptide complexes. While sequence information is informative, the exclusion of structural details may limit the accuracy and coverage of predictions, particularly for cases where structural features play a crucial role. Even some tools consider structure information, they seldom consider the structure features at the amino acids level. Besides, the length difference between the peptides that HLA can bind (typically around 8–15 amino acids) and the length of HLA molecules (which can be over 360 amino acids) poses a challenge in HLA-peptide binding affinity prediction. Furthermore, unlike HLAs, peptides are too short to form stable structures. All these drawbacks are not well solved by existing methods.

Considering all these limitations, we proposed GIHP, which is an interpretable GCNN-based algorithm for the prediction of peptides binding to pan HLA molecules. By representing peptide SMILE strings (Quiros et al., 2018; Meng et al., 2024) and HLA structures as attributed graphs, GCNNs can effectively model the pairwise interactions between amino acids and capture both local and global structural features. Furthermore, GIHP has a novel visual explanation method called Grad-WAM for HLA-peptide binding affinity prediction and interpretation. By analyzing the learned representations and interactions within the graph structure, the Grad-WAM technique can identify the key residues that contribute most significantly to the HLA-peptide binding process. Comprehensive comparative evaluation results demonstrate that the GIHP achieves good performance across diverse benchmark datasets. By applying the GIHP framework to several cancer immunotherapy datasets, we have identified numerous promising biomarkers that can effectively distinguish patients with and without treatment response. Moving forward, the insights gained from the GIHP analysis can be leveraged to guide the development of more personalized cancer immunotherapy strategies.

## 2 Materials and methods

### 2.1 Data collection and processing

We collected human HLA-peptide interaction datasets from published papers or publicly available databases. (Table 1).

**TABLE 1:** Summary of the collected datasets after preprocessing.

Name	HLAs	Peptides	HLA-peptide interactions
Wang-2008	26	4,421	24,295
Wang-2010	14	3,902	9,478
Kim-2014	183	28,428	268,189
Jurtz-2017	124	3,307,868	3,618,591
Jensen-2018	72	15,965	131,008
Zhao-2018	53	2,168	21,092
Reynisson-2020	161	4,523,148	4,795,633

Wang-2008 Dataset (Wang et al., 2008): Experimentally measured peptide binding affinities for HLA class II molecules. The processed data set had 24,295 interaction entries in total with ligand length ranging from 16 to 37 and have 26 unique HLA molecules. HLA DP and DQ molecules are covered.

Wang-2010 Dataset (Wang et al., 2010): Experimentally measured peptide binding affinities for MHC class II molecules. After preprocessing, the dataset contains 9,478 measured affinities and covers 14 MHC class II alleles with peptides length ranging from 9 to 37.

Kim-2014 Dataset (Kim et al., 2014): this dataset was obtained from the Immune Epitope Database (IEDB) (Vita et al., 2019), including binding affinity data compiled in 2009 (BD 2009), 2013 (BD 2013) and also include a blind datasets. Blind datasets refer to data resulting after subtracting BD2009 from BD 2013. For all these three datasets, only human datasets were kept for training. After preprocessing the dataset contains 268,189 interactions in total, with peptides length ranging from 8 to 30.

Jurtz-2017 Dataset (Jurtz et al., 2017): this dataset is originally designed for training of NetMHCPan-4.0. The final processed dataset has 3,618,591 entries in total with ligand length ranging from 8 to 18.

Jensen-2018 Dataset (Jensen et al., 2018): this dataset is used for training of NetMHCIIpan-3.2 (Karosiene et al., 2013), which contains HLA class II binding affinities retrieved from the IEDB in 2016. The 2016 data set contains 131,008 data points, covering 36 HLA‐DR, 27 HLA‐DQ, 9 HLA‐DP molecules and 15,965 unique peptides. The peptides length range from 9 to 33.

Zhao-2018 Dataset (Zhao and Sher, 2018): this dataset is compiled for training IEDB tools as well as the MHCflurry (O'Donnell et al., 2018). The dataset contains 21,092 binding relations, covering 18 HLA‐DR, 19 HLA‐DQ, 16 HLA‐DP molecules and 2,168 unique peptides. The peptides length is 15.

Reynisson-2020 dataset (Reynisson et al., 2020): this dataset is originally collected for training NetMHCpan-4.1 and NetMHCIIpan-4.0 methods. The dataset covering 161 distinct HLA class I molecules, 4,523,148 distinct peptides, with peptides length ranging from 8 to 15.

For all the collected training datasets, only binding affinity values in IC50nM format are kept, which are log‐transformed to fall in the range between 0 and 1 by applying 1−log (IC50 nM)/log (50k) as explained by Nielsen et al. (2003). When classifying the peptides into binders or non-binders a threshold of 500 nM is used. This means that peptides with log50k transformed binding affinity values greater than 0.426 are classified as binders. We consolidated all the collected datasets, removing any duplicate entries, to arrive at a final integrated dataset comprising 160,253 unique HLA-peptide interactions, covering 223 distinct HLA alleles and 35,481 peptide sequences. To further verify the generality of our method, we collected protein-peptide binding data from pepBDB (Wen et al., 2019) database, after deleting peptides short than 8aa, we got 12,655 interactions between 11,055 proteins and 7,811 peptides. Because our method takes HLA and protein structure as input, all the structure data are downloaded from the PDB (Berman et al., 2000) and AlphaFold database (Varadi et al., 2022) and some are predicted by alphafold2 (Jumper et al., 2021) and Rosettafold (Baek et al., 2021). Only high-resolution experimental structures (e.g., X-ray crystallography or cryo-EM data with resolution better than 3.0 Å) were included. All structural models, whether experimental or predicted, were subjected to validation using atomic contact evaluation, and overall model quality assessment. Only structures that passed these validation checks were retained for further analyses.

To evaluate whether the key binding residues identified by our method can effectively differentiate patients who benefit from immunotherapy, we collected relevant breast, bladder, and pan-cancer treatment datasets from the cBioPortal resource (Cerami et al., 2012), as shown in Table 2. Key binding residues mutation could lead to binding affinity change between HLA and peptides. Binding affinity change has been demonstrated as a biomarker of immunotherapy efficiency (Kim et al., 2020; Seidel et al., 2021; Murata et al., 2022). For each patient, only SNP mutations are kept, if the SNP locates on the key binding site of HLA or peptide, then we separate them in one group, otherwise in the other group. Then we conduct survival analysis for the two groups.

**TABLE 2:** Immunotherapy related dataset and three cancer datasets.

Name	Type	Patients	SNP mutations
Samstein-2019	Pan-cancer	1,662	14,876
Miao-2018	Pan-cancer	249	102,207
Razavi-2018	Breast cancer	1,756	7,420
Clinton-2022	bladder Cancers	1,245	24,277
Aaltonen-2020	Pan-cancer	2,583	347,994

Samstein-2019 dataset (Samstein et al., 2019): The cohort consisted of 1,662 patients, received at least one dose of immune checkpoint inhibitor (ICI) therapy. The cohort encompassed a variety of cancer types with an adequate number of patients for analysis. In detail, 146 patients received anti-CTLA4, 1,447 received anti-PD1 or PD-L1, and 189 received both. This is a pan-cancer dataset, including 350 cases of non-small cell lung cancer (NSCLC), 321 cases of melanoma, 151 cases of renal cell carcinoma (RCC), 214 cases of bladder cancer, and 138 cases of head and neck squamous cell cancer.

Miao-2018 dataset (Miao et al., 2018): this dataset consists of 249 patient tumors from six different cancer types: melanoma (*N* = 151), non-small cell lung cancer (*N* = 57), bladder cancer (*N* = 27), head and neck squamous cell carcinoma (*N* = 12), anal cancer (*N* = 1), and sarcoma (*N* = 1). These patients were treated with anti-PD-1 therapy (*N* = 74), anti-PD-L1 therapy (*N* = 20), anti-CTLA-4 therapy (*N* = 145), or a combination of anti-CTLA-4 and anti-PD-1/L1 therapies (*N* = 10). A small proportion of patients (*N* = 7) received a combination of anti-PD-1, anti-PD-L1, or anti-CTLA-4 therapy with another immunotherapy, targeted therapy, or cytotoxic chemotherapy.

Razavi-2018 dataset (Razavi et al., 2018): This dataset is downloaded from cBioPortal: https://cbioportal-datahub.s3.amazonaws.com/breast_msk_2018.tar.gz.

Clinton-2022 dataset (Clinton et al., 2022): This dataset is downloaded from cBioPortal: https://cbioportal-datahub.s3.amazonaws.com/paired_bladder_2022.tar.gz.

Aaltonen-2020 dataset (Consortium et al., 2020): This dataset is downloaded from cBioPortal: https://cbioportal-datahub.s3.amazonaws.com/pancan_pcawg_2020.tar.gz.

### 2.2 Methods

**FIGURE 1:**
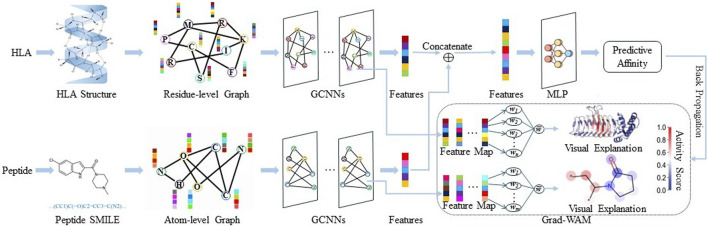
The overall framework of GIHP.

The overall framework of GIHP is illustrated in Figure 1. GIHP takes HLA structure and peptide SMILE string as input. In the input representation module, HLA is represented as an attributed residue-level graph, while the peptide is represented as an attributed atom-level graph. Then a multi-layer GCNNs is used to learn the high-level features, and the learned features are contacted and fed into the MLP layer for final binding affinity prediction. To enhance the results interpretability, we introduced a novel visual interpretation method called Grad-WAM. Grad-WAM leverages gradient information from the last GCN layer to assess the significance of each neuron in determining affinity.

#### 2.2.1 Input representation

Graph-based protein structure representation has inherent advantages over traditional sequence-based approaches in capturing true binding events. For each HLA molecular, we take both structure and sequence information into consideration. Given one of our key objectives is to identify the critical binding amino acid residues, we have represented the HLA proteins as residue-level relational graphs $G_{H}=\left(v,\varepsilon \right)$ , where v is the set of amino acids, ε is the set of edges. As shown in Table 3, we describe the node attributes by integrating sequence and structural property, including amino acid type, chemical properties, charges, *etc.*, while the edge attributes encompass connection types, distances, and structural information. We consider four types of bond edges including Peptide Bonds, Hydrogen Bonds, Ionic Bonds and Disulfide Bridges.

**TABLE 3:** The node features of HLA graph.

Name	Description	Dim
Residue type	We utilize Blosum62, 20 types of amino acids plus 1 unknown	21
Structure mapping	Included α-helix (H), residue in isolated β-bridge (B), extended strand, participates in β ladder (E), hydrogen bonded turn (T),3_10_ helix (G), π-helix (I), bend (S) and coil (C)	8
AA position	the position of α-carbon in each residue to record their 3D position	3
Hydrogen donor or acceptor	Donor: R, K, W. Acceptor: D, E. Donor and acceptor: N, Q, H, S, T, Y	4
Physicochemical properties	We utilize a set of 7 physicochemical properties for amino acid types (AAPHY7). These features include steric parameters, hydrophobicity, volume, polarizability, isoelectric point, helix probability, and sheet probability	7

Considering that the length of peptides binding to MHC class II is between 13–25 residues, and the length is around nine for peptides binding to MHC class I. Therefore, the peptide length is relatively short compared to HLAs (over 360aa). In this study, we represent peptides as SMILES-like sequences and then transform them into graphs using a molecular graph representation method inspired by RDKit (https://www.rdkit.org). The attributes of each node $v_{i}$ are shown in Table 4. $e_{ij}\in \varepsilon$ is covalent bonds between the *ith* and the *jth* atoms. The edge attributes depending on the electrons shared between atoms, resulting in single, double, or triple bonds, respectively.

**TABLE 4:** Node features of peptide graph.

Name	Description	Dim
Atom type	[H, C, N, O, F, Cl, S, Br, I] (one-hot)	9
Atomic Num	The atomic number (integer)	1
Acceptor	Accepts electrons [0/1] (binary)	1
Donor	Donates electrons [0/1] (binary)	1
Aromatic	In an aromatic system [0/1] (binary)	1
Hybridization	[sp, sp2, sp3] (one hot)	3
Hydrogens	Number of connected hydrogens (integer)	1
Formal charge	Formal charge of the atom (integer)	1
Explicit valence	Explicit valence of the atom (integer)	1
Implicit valence	Implicit valence of the atom (integer)	1
Explicit Hs	Number of implicit Hs the atom is bound to (integer)	1
Radical electrons	Number of radical electrons for the atom (integer)	1

#### 2.2.2 Graph convolutional neural network module

Let A be the adjacency matrix, and X be the feature matrix of the given graph. Each GCN layer takes A and node embeddings as input and outputs the final embeddings. As shown in Eqs 1, 2.

$$H^{\left(l+1\right)}=GCN\left(H^{\left(l\right)},A\right)$$ (1)

$$H^{\left(l+1\right)}=ReLU\left({\hat{D}}^{-0.5}\hat{A}{\hat{D}}^{-0.5}H^{\left(l\right)}W^{\left(l+1\right)}\right)$$ (2)

Where, H is the embeddings, and $H^{\left(0\right)}=X$ , $W^{\left(l+1\right)}$ are trainable weight matrix, $\hat{D}$ is the diagonal node degree matrix of A .

After obtaining the vector representations of HLA and peptide, they are concatenated and fed into a Multi-Layer Perceptron (MLP) to predict the binding affinity score. The MLP consists of three linear transformation layers, each followed by a Rectified Linear Unit (ReLU) activation function and a dropout layer with a dropout rate of 0.1, as in (Öztürk et al., 2019). The Mean Squared Error (MSE) is employed as the loss function to measure the discrepancy between predicted and actual affinity scores. MSE is defined in Eq. 3.

$$MSE=\frac{1}{n}\sum_{i=1}^{n}{\left(P_{i}-Y_{i}\right)}^{2}$$ (3)

Where, n is the sample size, $P_{i}$ and $Y_{i}$ are the predictive and true values of the *ith* interaction pair, respectively.

#### 2.2.3 Gradient-weighted activation mapping

While Grad-CAM has been successfully applied to various computer vision tasks, it is not directly applicable to graph-structured data. Therefore, in this paper we proposed a novel results interpretation methods called Grad-WAM, which can be used for identifying key binding related residues. Grad-WAM measure the contribution of each residue for the decision of binding by taking use of the gradient information in the last GCN layer. Grad-WAM utilizes a weighted combination of the positive partial derivatives of the feature maps with respect to the interaction values to generate the corresponding visual explanations. Considering the contribution of each residue is not equal, different from the explanation method proposed in MGraphDTA (Yang et al., 2022), we introduce an additional weight ω (Eq. 4) gradient values.

$$\omega =\sum_{i}\left[\alpha_{i}\right]\cdot ReLU\left(\frac{\partial P}{\partial T_{i}}\right),\forall \left\{i\;|\;i\epsilon T\right\}$$ (4)

Where, ReLU is the activation function, P is the predictive value as in Eq. 5. $T_{i}$ is the feature value of the *ith* node on the feature map T of the last GCN layer. $\alpha_{i}$ is the gradient value of the *ith* node defined in Eq. 6. $\frac{\partial P}{\partial T_{i}}$ is the partial derivative as in Eq. 7.

$$P=\sum_{i}\alpha_{i}\cdot ReLU\left(\frac{\partial P}{\partial T_{i}}\right)\cdot T_{i}$$ (5)

$$\alpha_{i}=\frac{\frac{\partial P}{\partial T_{i}}}{\frac{\partial P}{\partial T_{i}}+T_{i}\cdot \frac{\partial^{2}P}{{\left(\partial T_{i}\right)}^{2}}\;}$$ (6)

$$\frac{\partial P}{\partial T_{i}}=\alpha_{i}\cdot \frac{\partial P}{\partial T_{i}}+T_{i}\cdot \alpha_{i}\cdot \frac{\partial^{2}P}{{\left(\partial T_{i}\right)}^{2}}$$ (7)

In this way, the contribution of residues to the prediction of binding affinity is calculated. For visual explanation, residues are display utilizes colors, ranging from blue to red. A higher gradient value corresponds to a redder color, indicating the key role of that amino acid in the interaction.

## 3 Results

### 3.1 Performance comparisons with other methods

Four widely used performance metrics were employed to measure methods’ performance. Including accuracy (*Acc*), Matthews Correlation Coefficient (*MCC*), sensitivity (*Sn*), and the specificity (*Sp*). The definitions of these four metrics are as follows: Eqs 8–11.

$$Acc=\frac{TP+TN}{TP+TN+FP+FN}$$ (8)

$$Sn=\frac{TP}{TP+FN}$$ (9)

$$Sp=\frac{TN}{TN+FP}$$ (10)

$$MCC=\frac{TN\times TP-FN\times FP}{\sqrt{\left(TP+FP\right)\left(TP+FN\right)\left(TN+FP\right)\left(TN+FN\right)}}$$ (11)

Where, *TP* is True Positives, *TN* is True Negatives, *FP* is False Positives, and *FN* is False Negatives. In addition, by comparing the predicted and true values, predictions were assessed to be true or false. The receiver operating characteristic curves (ROC) were generated for all the methods, and the performance of each algorithm to discriminate between binders and nonbinders was analyzed by calculating the area under the ROC curve (AUC) as an estimate of prediction performance.

**FIGURE 2:**
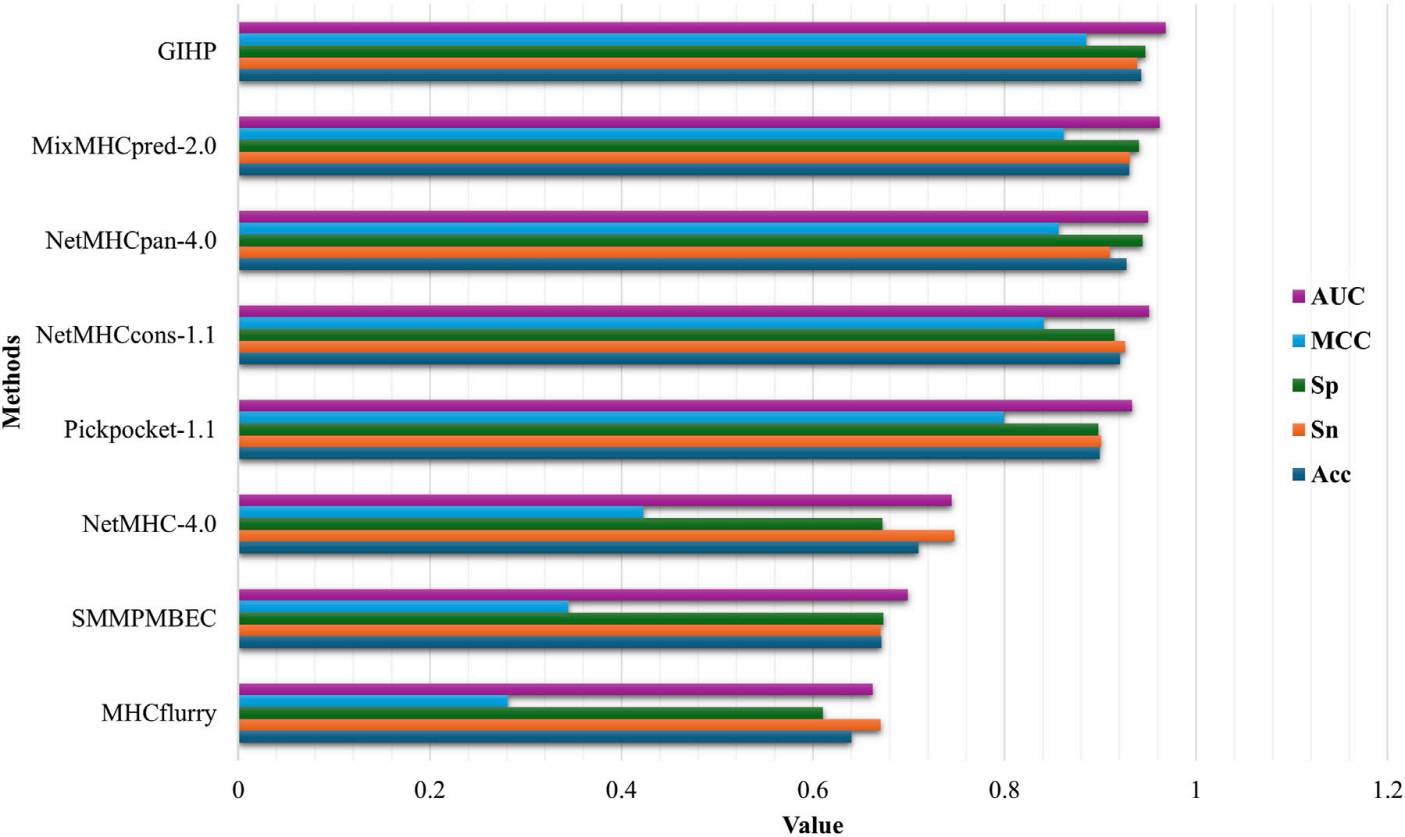
Performance comparison results.

We compare GIHP with state-of-the-art allele and pan-specific baselines including NetMHC-4.0 (Andreatta and Nielsen, 2016), NetMHCpan-4.0 (Jurtz et al., 2017), PickPocket-1.1 (Zhang et al., 2009), SMMPMBEC (Kim et al., 2009), MHCFlurry (O'Donnell et al., 2018), MixMHCpred-2.0 (Bassani-Sternberg et al., 2017) and NetMHCcons-1.1 (Karosiene et al., 2012). To eliminate the impact of data variations, all models were retrained and tested using our new collected and processed dataset. 10-fold cross-validation (CV) was applied. The data set is divided into 10 folds. During each iteration, one of the 10 partitions is designated as the validation dataset, while the remaining nine partitions are utilized to train the model. The final performance is determined by calculating the average performance across all 10 individual iterations. As shown in Figure 2, on average, GIHP outperform all the compared prediction methods. It is worth noting that not every method is suitable for every HLA and peptide length. To make the performance comparison fairer and more reasonable, we train allele-specific models with their required HLAs and peptide length, which included in our datasets.

**FIGURE 3:**
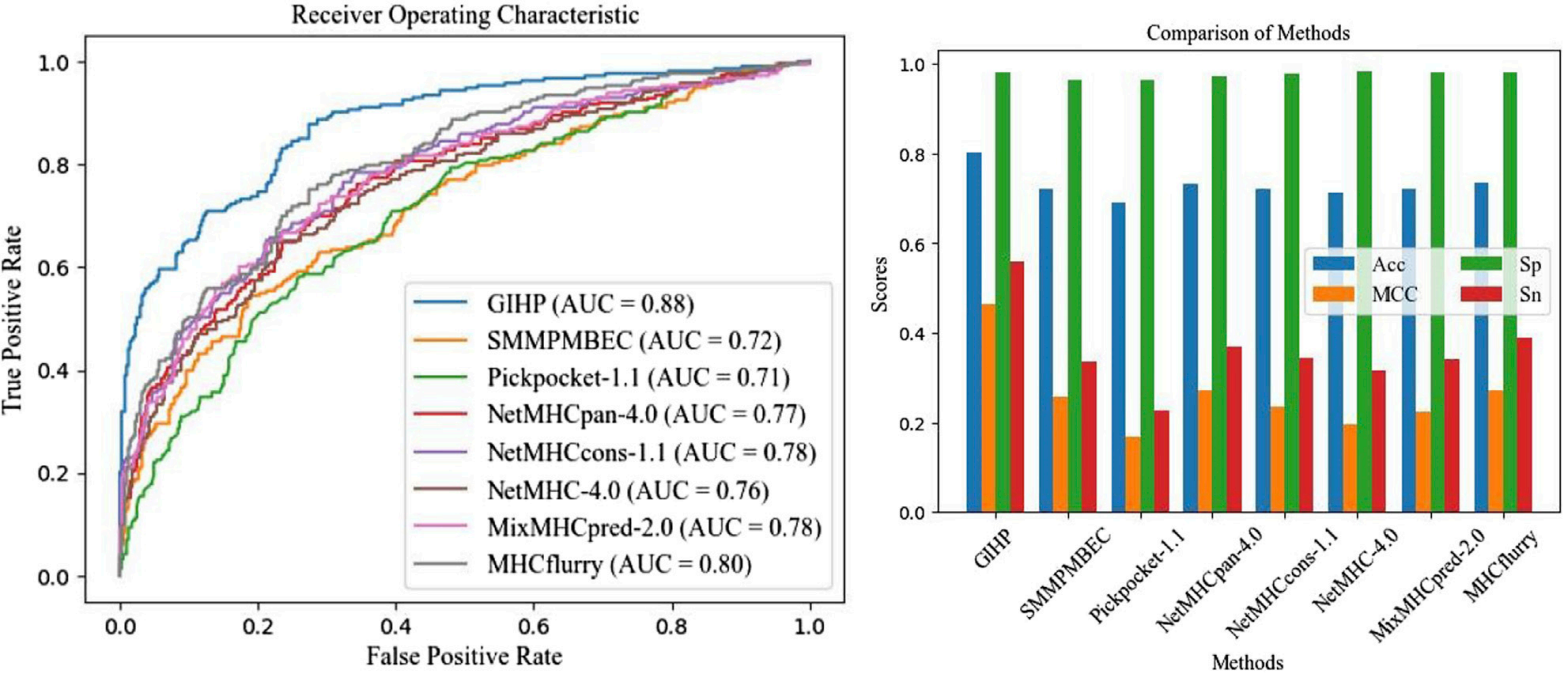
Independent test results on pepBDB datasets.

To make comparisons more comparable and test methods performance on other protein-peptide binding datasets, a separate independent test is conducted using the data collected from pepBDB, which have no overlap with the above training data. This independent test data set serves as an unbiased validation source to assess the performance of different tools, which is relatively more objective, and can test models’ generalization ability. 10-fold cross validation is applied, after each epoch average results are calculated. Results on the pepBDB independent test data is shown in Figure 3.

On average, GIHP achieved highest AUC value. In this independent test data, GIHP achieved the highest AUC of 0.88 and the highest Sp score of 0.98. In contrast, NetMHCPan-4.0 and Pickpocket-1.1 attained AUC values of 0.76 or lower, and Acc scores of 0.71 or lower when evaluated on this new dataset. Difference from the results on the above part, MHCflurry got AUC up to 0.8. Similar with our method, MHCflurry harness the power of deep learning and a comprehensive dataset to improve the prediction of HLA-peptide binding affinities. Our model outperforms both allele and pan-specific methods, demonstrate its ability to achieve higher prediction accuracy and robustness generality for all kinds of training data.

**FIGURE 4:**
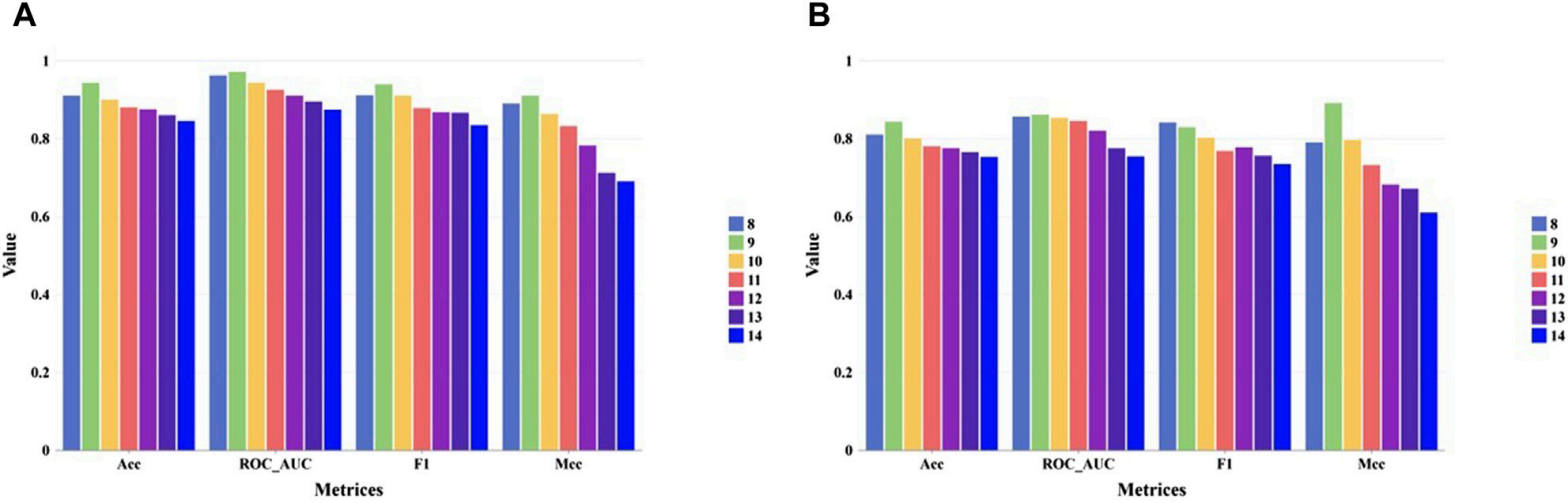
The performance of our model on the independent test set and external test set for the different peptide lengths. **(A)** Performance on the independent test set. **(B)** Performance on the external tet set.

For evaluating the performance our method under different peptide length. We collected independent test set and external test set from TransPHLA, which can be downloaded from https://github.com/a96123155/TransPHLA-AOMP/tree/master/Dataset. In the collected datasets, 9-mer peptides comprising the largest proportion, while the number of 13-mer and 14-mer peptides is very small. Our model’s performance on the independent test set and external test set for different peptide lengths are shown in Figures 4A, B respectively. As shown in Figure 4, our methods can achieve good performance on all kinds of peptide length.

### 3.2 Key binding residues on HLAs

**FIGURE 5:**
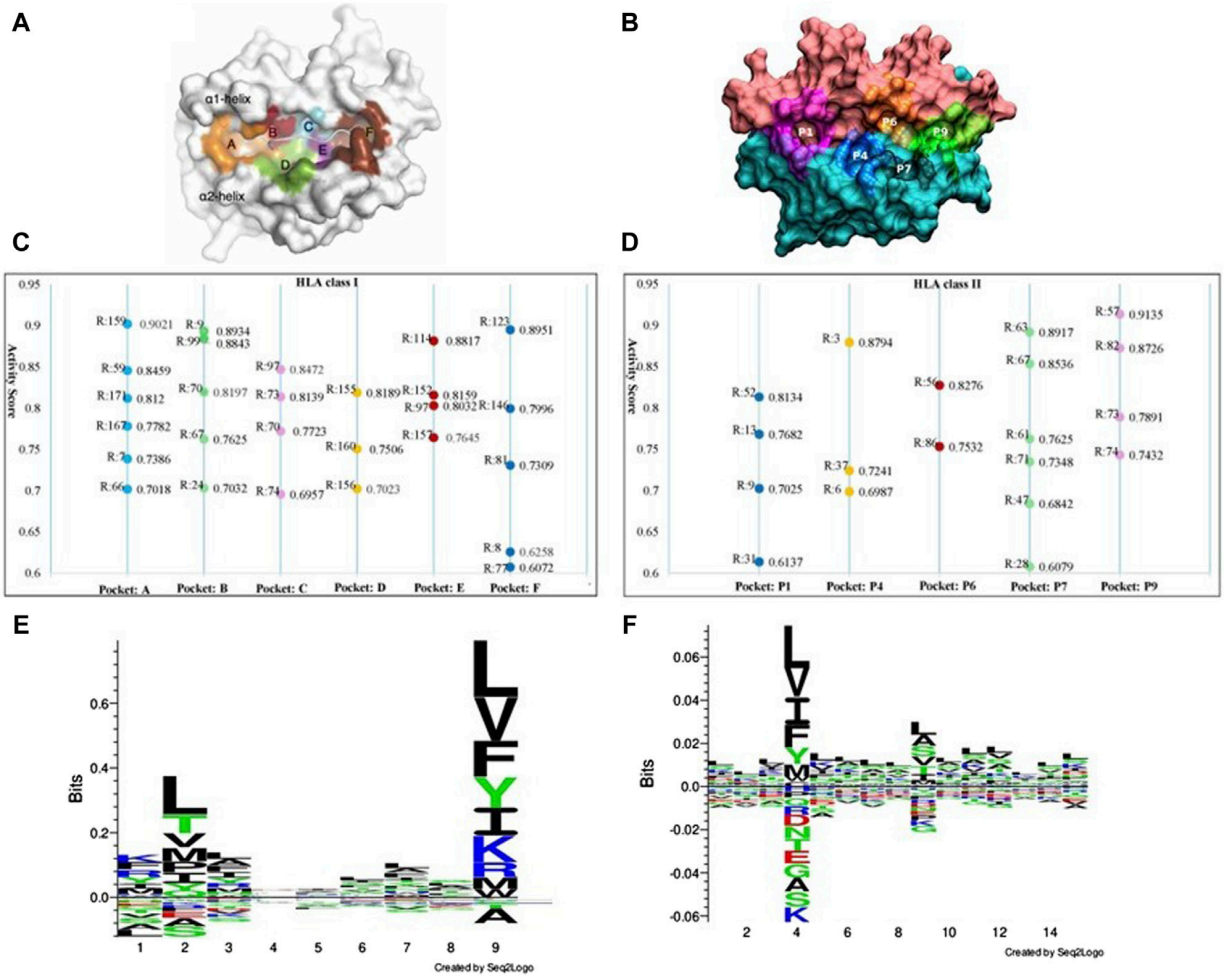
The key binding residues on HLA pockets and HLA binding peptides motif. **(A)** Binding pockets on HLA class I molecules. **(B)** Binding pockets on HLA class II molecules. **(C)** The identified key binding residue locations and activity scores on each pocket of HLA class I molecules, where R represent residue location analyzed HLA molecules. **(D)** The identified key binding residue locations and activity scores on each pocket of HLA class II molecules. **(E)** Distribution of preferred peptide residues of HLA class I molecules using Seq2logo2.0. **(F)** Distribution of preferred peptide residues of HLA class I molecules using Seq2logo2.0.

The binding of peptides to HLA molecules occurs within specialized regions called binding pockets. HLA class I molecules have a peptide-binding groove formed by two alpha helices (α1 and α2) and a beta sheet platform. Within this groove, there are seven pockets (numbered from A to F, shown in Figure 5A) that interact with specific amino acid residues of the bound peptide. HLA class II molecules are involved in presenting peptides derived from extracellular proteins to helper T cells. HLA class II binding pockets are formed by two chains: the alpha chain (α) and the beta chain (β). Each chain consists of two domains: the α1 and β1 domains form the peptide-binding groove, while the α2 and β2 domains provide structural support. The binding groove of HLA class II molecules is open at both ends, allowing longer peptides to bind compared to HLA class I molecules. The binding pockets in HLA class II molecules are referred to as P1, P4, P6, P7, P9 (Figure 5B). With our GIHP results interpret module, many key binding residues on both HLA class molecules and the corresponding peptides are identified. Although some residues with high activity scores locates outside of binding pockets, most of them locates on one of the binding pockets. As shown in Figures 5C,D, 45 residues with highest activity scores on HLAs are identified, among them 26 locates on HLA class I pockets, and 19 locates on HLA class II pockets.

Position 159 has the highest activity score on pockets A. Other positions including 59, 171, 167, seven and 66. According to current research, position seven is a pocket A’s floor residue. This residue creates a hydrophobic environment within the pocket A and interact with the side chain of the anchor residue. Although residue on position 159 has no evidence of directly involved in peptide binding interactions, it has structural and functional implications for the overall stability and conformation of the pocket A region (Ma et al., 2020). It potentially contributes to the shape and electrostatic properties of the pocket, indirectly affecting the binding preferences and stability of the peptides presented by the HLA class I molecule. However, the specific role and impact of residue 159 on the pocket A’s function vary among different HLA alleles and need further study for a comprehensive understanding. On pockets B, substitutions at positions 70 was found to yield a significantly distinct peptide-binding repertoire in HLA-A molecules when compared to HLA-B molecules. Positions 167 and position 67 on pocket B has been demonstrated as key peptide-binding residues. Besides, substitutions at positions 67 and nine exert a significant influence on the peptide-binding repertoire (van Deutekom and Keşmir, 2015). Position 97 has the highest activity score on pockets C. Position 97 is known to be a critical residue for peptide binding and presentation. This residue locates near the C-terminal anchor residue of the bound peptide and contributes to the formation of the peptide-binding groove. The amino acid at position 97 can significantly influence the peptide-binding specificity and affinity of the HLA molecule. Substitutions or variations at this position can alter the size, shape, or electrostatic properties of the pocket C, thereby affecting the recognition and binding of specific peptides. Several studies have investigated the impact of position 97 on peptide binding and immunological responses (Moutaftsi et al., 2006).

Considering the residues with high activity scores on HLA class II pockets, position nine is crucial for determining the peptide-binding specificity of the HLA class II molecule. The amino acid at position nine of the bound peptide interacts with residues in the P1 pocket, influencing the peptide-binding preferences. Position 86 plays a critical role in peptide binding and presentation (Brown et al., 1993). The amino acid at position 86 interacts with the peptide residue and contributes to the stability and specificity of the HLA-peptide class II complex (Stern et al., 1994). Among our identified important positions, positions 13 and 74 are critical for determining the peptide-binding specificity and stability of HLA class II molecules. The interactions between peptide residues and the residues in these pockets are essential for the recognition and presentation of antigenic peptides to CD4^+^ T cells. Except these positions, we also prioritized many other residues, such as positions 63 and 57. These positions within the peptide-binding grooves of HLA class II molecules is crucial for understanding the molecular basis of antigen presentation and immune responses. Researchers can gain valuable information about the molecular interactions governing antigen presentation and T cell recognition. Furthermore, these results can help designing personalized immunotherapies (Boukouaci et al., 2024).

Figures 5E, F show the motif analysis results. In the two figures, the Y-axis describes the amount of information in bits. The X-axis shows the position in the alignment. At each position there is a stack of symbols representing the amino acid. Large symbols represent frequently observed amino acids, big stacks represent conserved positions and small stacks represents variable positions. Therefore, positions 2, 4 and nine have frequently observed amino acids in HLA class I and class II respectively.

### 3.3 Key binding residues on peptides and their corresponding genes

**FIGURE 6:**
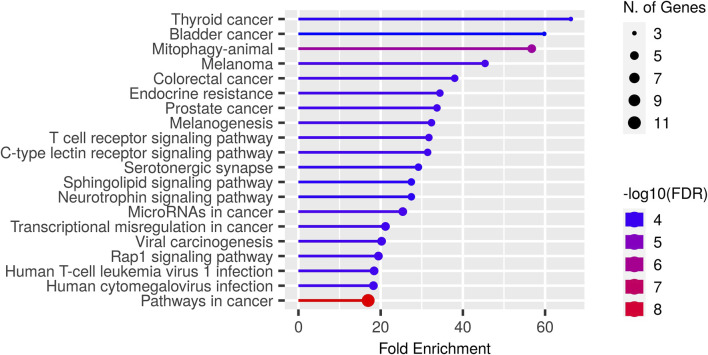
GO enrichment results of key residues related genes.

In this paper, we focus on finding immunotherapy efficiency related key residues and their corresponding genes. With the identified residue positions and the corresponding gene mutation, we try to verify whether they can be biomarkers to separate patients into different survival groups. We applied GIHP to immunotherapy related datasets (Samstein-2019 and Miao-2018 in Table 2). For each SNP mutation site, we extract the corresponding 9-mer peptide around it and predict the binding affinities with all the 223 HLAs. By paired *t*-test statistical comparing the binding affinity change before and after residue substitution, along with GIHP returned activity scores of each residue, significant key binding residues are identified. To get the functions of these mutation related genes, we conducted GO enrichment analysis by ShinyGO-0.80 (Ge et al., 2020). As shown in Figure 6, most of key residues locate on genes related to pathways in cancer and cancer related signaling pathways.

Since we interest in finding mutations related to immunotherapy response, therefore, we further analyzed key residues enriched in T cell receptor signaling pathway (Figure 6). The enriched genes include RHOA, HLA-B, HRAS, IL10, NRAS and KRAS. RHOA has been implicated in T cell activation and migration, which are critical for effective anti-tumor immune responses (Bros et al., 2019). Altered RHOA signaling could potentially impact T cell function and infiltration into the tumor microenvironment, influencing immunotherapy response. HLA-B plays a crucial role in immune recognition, as it presents peptide antigens derived from intracellular proteins to cytotoxic T cells. HRAS, NRAS, and KRAS are genes that belong to the RAS family of oncogenes. These genes encode proteins involved in intracellular signaling pathways regulating cell growth, survival, and proliferation. The presence of RAS mutations has been associated with poorer response rates to certain immunotherapies, including immune checkpoint inhibitors (East et al., 2022). IL10 can suppress the activity of cytotoxic T cells and natural killer (NK) cells, which are critical for tumor surveillance and elimination. High levels of IL10 in the tumor microenvironment have been associated with immunosuppression and reduced response to immunotherapy (Salkeni and Naing, 2023).

**FIGURE 7:**
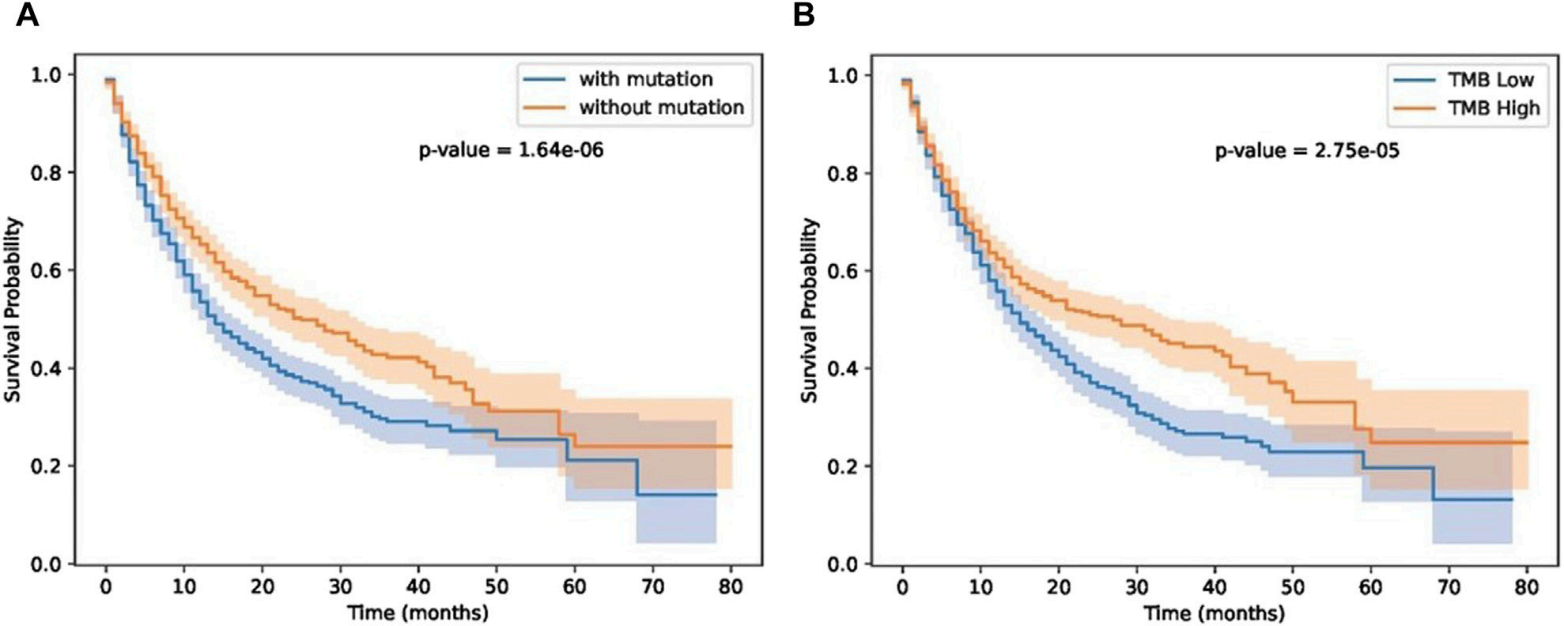
Results on immunotherapy data. **(A)** patient groups separated by GIHP identified biomarker mutations. **(B)** TMB separated patient groups.

Next, we investigated the impact of biomarker gene mutations on patient survival outcomes using a cohort of individuals (Samstein-2019 dataset in Table 2) with immunotherapy treatment. The patients were categorized into two groups based on the presence or absence of the biomarker gene mutation. Kaplan-Meier survival curves were generated, and a log-rank test was performed to compare the survival between the two groups. The results revealed a significant difference in survival between the two groups, with patients harboring the biomarker gene mutation exhibiting a higher risk of adverse events compared to those without the mutation. These findings highlight the potential prognostic significance of the biomarker gene mutation and underscore its relevance in patient stratification and personalized treatment approaches. Furthermore, we compared our results with TMB score provided in (Samstein et al., 2019). As shown in Figure 7, patients with biomarker mutations tend to have poor survival status.

**FIGURE 8:**
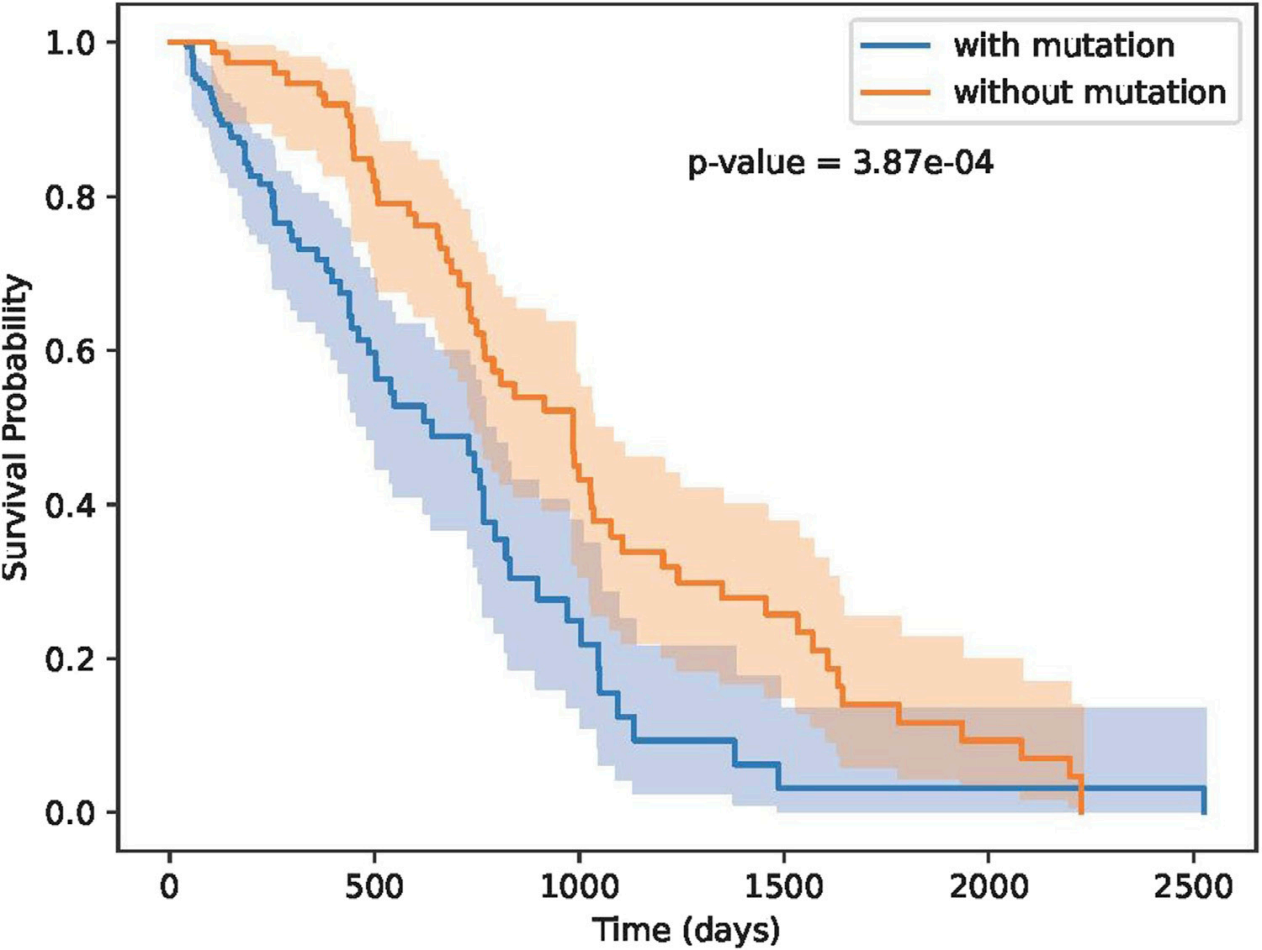
Results on Miao-2018 datasets.

As shown in Figure 7, our methods can separate patients more significantly. Although TMB can separate patients, TMB is an overall measure, its hard to know which gene mutations play key roles in differentiating patients’ response. Our methods not only can separate patients significantly, moreover, we also know which residue substitutions play key roles. To further test the performance of these biomarker genes, we analyzed Miao-2018 datasets (Table 2), results is show in Figure 8.

As illustrated in Figure 8, the identified biomarker mutations are also able to effectively separate patient groups with statistical significance. Our findings provide compelling evidence that the identified biomarker genes may possess valuable predictive power for immunotherapy response and patient survival outcomes. This highlights their potential as clinically relevant targets for the development of personalized treatment approaches. The results of this study advance the understanding of the underlying molecular mechanisms governing immunotherapy efficacy, and offer promising directions for future research and therapeutic interventions.

### 3.4 Performance on other cancer datasets

**FIGURE 9:**
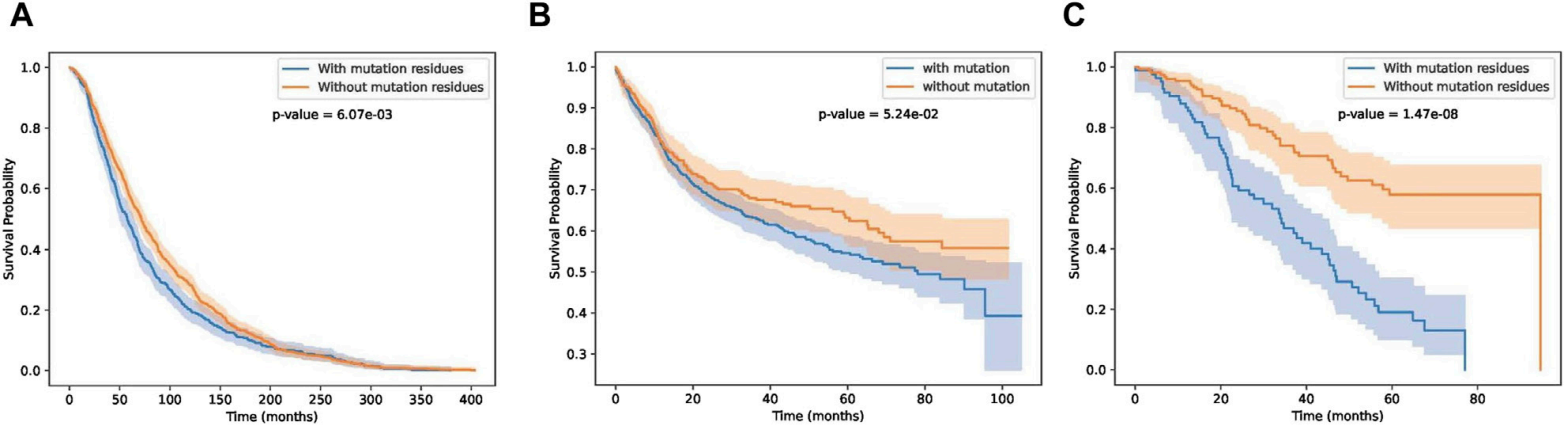
Survival curves on breast, bladder and pan cancer datasets.

In this section, we test whether these key residue mutations and their corresponding genes can separate other cancer patients. Results are shown in Figures 9A–C. Detail information of these three cancer datasets are shown in Table 2. We can see that our biomarker genes can differentiate the three-cancer type significantly. Especially for the pan cancer datasets.

## 4 Conclusion

In summary, we proposed a new GCNN-based framework called GIHP for pan-specific HLA-peptide binding affinity prediction. GIHP harness both structure and sequence information and utilized Grad-WAM for visual interpretation. Extensive comparison with state-of-the-art methods verified the better performance of our methods. Collectively, the findings provide evidence that the GIHP framework has improved the generalization and interpretability capabilities of HLA-peptide binding prediction models. Furthermore, we have identified numerous key binding-related amino acid residues that can serve as potential biomarkers for differentiating patient groups based on immunotherapy response. When applying these identified biomarkers on datasets from other cancer types, they were also able to effectively differentiate patient groups with statistical significance. These findings highlight the potential prognostic significance of the biomarker gene mutation and underscore its relevance in patient stratification and personalized immunotherapy treatment approaches.

## Data Availability

The data presented in the study are deposited in the Github, accession link: https://github.com/sdustSu/GIHP.
